# What’s an Architect Doing in a Place Like This?…My Journey From Form to Function to Facilitate Aging in Place

**DOI:** 10.1093/geroni/igaf122.337

**Published:** 2025-12-31

**Authors:** Jon Sanford

**Affiliations:** Georgia State University, Atlanta, Georgia, United States

## Abstract

My journey in evidence-based, user-centered design to promote activity performance and aging in place started long before these were clinical buzzwords. With an undergraduate degree in social psychology, I recognized early on in my architecture education that there is an inherent disconnect between architects’ assumptions about human needs, preferences, and abilities and the people they design for. As luck would have it, Georgia Tech had one of the earliest programs in environment and behavior research, and funding for accessibility and falls research put me through graduate school. This was followed by my own research funding (which also created a faculty position) for the early accessibility standards. Soon after, I moved on to the new Atlanta VA Rehab R&D Center on Geriatric Rehabilitation, where I was one of 3 research architects in the VA system. At the same time, I helped start the Center on Universal Design at NC State, where I co-authored the Principles of Universal Design. I also secured funding for a Chicago aging-in-place remodeler to develop one of the earliest remote home modifications programs. Then, it was back to GA Tech at the Center for Assistive Technology and Environmental Access (CATEA), where I was a professor of industrial design and one of the three founding PIs of RERC TechSAge. The one commonality in each project along the journey has been my collaboration with occupational therapists (OTs). With this in mind, it is easy to see what an architect is doing as the Research Director in an OT program.

